# Oxidative Stress and Membrane Transport Systems

**DOI:** 10.1155/2018/9625213

**Published:** 2018-06-13

**Authors:** Angela Marino, Silvia Dossena, Grazia Tamma, Sandra Donnini

**Affiliations:** ^1^Department of Chemical, Biological, Pharmaceutical and Environmental Sciences, University of Messina, Messina, Italy; ^2^Institute of Pharmacology and Toxicology, Paracelsus Medical University, Salzburg, Austria; ^3^Department of Biosciences, Biotechnologies and Biopharmaceutics, University of Bari “Aldo Moro”, Bari, Italy; ^4^Department of Life Sciences, University of Siena, Siena, Italy

Abnormal intracellular levels of reactive species (RS), including oxygen and nitrogen RS, are recognized as common denominator of numerous pathological conditions including aging and frailty. In the recent years, it has been widely accepted that high levels of intracellular RS can be associated with several chronic disturbs, such as Alzheimer disease (AD), chronic kidney disease, atherosclerosis, and cardiovascular and endothelial dysfunctions.

Emerging concepts point out that RS can influence intracellular transduction pathways by acting as key signal molecules. Therefore, the roles of free radicals have been strongly redefined. Plasma membrane, as the interface between intracellular and extracellular environments, may sense several molecules, including RS, through the action of membrane receptors and channels that also mediate the transport of RS, such as hydrogen peroxide (H_2_O_2_) and nitric oxide (NO). In turn, membrane transport systems can also be controlled by oxidative signaling. In this respect, posttranslational oxidative modifications translate changes of oxidative intracellular environment under physiological and pathophysiological conditions.

This special issue aimed to depict a link between membrane transport systems and oxidative stress in health and disease. From the manuscripts received, we selected 5 reviews, 1 clinical study, and 1 research article that addressed the main objectives of this issue.

G. Tamma and coauthors reviewed the transport of RS as a novel function of the water channels aquaporins (AQPs). AQPs are widely expressed in the animal and plant kingdom, with 13 different isoforms found so far in mammals. AQPs are highly expressed in tissues displaying high water permeability, such as endothelia, kidney tubules, and secretory glands. However, AQPs are also expressed in skin and fat cells that, normally, are not characterized by relevant fluid transport. In this review, authors reported that AQPs facilitate RS transport in several cell types, including hepatocytes and endothelial cells. Special focus is given to the AQP3-mediated H_2_O_2_ transport, which plays an important role in modulating sperm mobility as well as cell migration, and AQP1-mediated NO transport, which is involved in vascular senescence. The link between AQP2, oxidative stress, and aging has been also reported. The emerging evidence of clinical implications deriving from RS abnormal permeation through AQPs is also emphasized.

K. Izuhara et al. reviewed the involvement of the anion transporter pendrin/SLC26A4 in inflammatory lung diseases, such as asthma and chronic obstructive pulmonary disease (COPD). In particular, the attention has been focused on the proinflammatory cytokine IL-13, a well-established marker of asthma. IL-13 stimulates the expression as well as the function of pendrin, which in turn facilitates thiocyanate (SCN^−^) transport into the airway surface liquid at the apical side of airway epithelium, where it reacts with H_2_O_2_, thereby producing hypothiocyanite (OSCN^−^) via the DUOX/peroxidase pathway. OSCN^−^ is involved in the innate defense of the lumen mucosa. Authors specifically focus on the different intracellular signal transduction pathways, that is, NF-*κ*B signaling and cell necrosis, activated by different OSCN^−^ concentrations. As such, the review not only highlights the involvement of pendrin in the pathogenesis of lung disease but, more importantly, also provides a useful basis to identify novel drug targets for oxidative stress-related lung disease.

The review proposed by S. G. Vitale and coauthors highlights the roles of oxidative molecules in the pathogenesis of endometriosis, a chronic disorder affecting about 10–15% of women during the reproductive period. Though the molecular basis of this disease is not completely clarified, several risk factors have been found. In this latter regard, the authors report about inflammatory signals and oxidative molecules concurring to endometriosis. Oxidative molecules alter the permeability of endothelial cells and the expression of adhesion proteins, thereby promoting inflammatory processes. Indeed, oxidative molecules can also modulate the function of different immune cells. Last but not the least, authors reported the involvement of reactive oxygen species (ROS) in modulating epigenetic processes that may increase the risk of ovarian cancer. Overall, the review underscores the crucial role of antioxidant treatment in mitigating endometriosis progression.

G. Sita and coauthors, in their review, discuss the involvement of ROS in the modulation of P-glycoprotein (ABCB1) expression levels. ABCB1 is an ATP-binding cassette transporter, playing a role in the pathogenesis of AD, the most common cause of dementia and mortality in elderly. This neurological disorder is associated with an abnormal accumulation of beta amyloid plaques in the extracellular environment, and the authors clearly define the contribution of oxidative molecules in promoting this adverse phenomenon. Several studies have been discussed in order to identify and clarify the intracellular signal transduction pathways controlled by ROS in AD.

The review article proposed by V. Fisi and coauthors focuses on the interplay between O-GlcNAc and oxidative stress in influencing membrane transport molecules. O-GlcNAc is a highly dynamic and abundant posttranslational modification that may compete with or influence phosphorylation and plays a fundamental role in controlling several physiological processes, such as insulin signaling, glucose transport, and stress adaptation. In addition, alterations of O-GlcNAc levels have been found in several chronic diseases such as AD, diabetes, and inflammation, suggesting that abnormally elevated or reduced O-GlcNAc levels may be a novel marker to predict the risk of degenerative diseases.

The clinical study performed by C. Torino and coauthors reported the effect of vitamin D receptor (VDR) stimulation on the advanced glycosylation end products receptor (AGE/RAGE) system in patients affected by chronic kidney disease (CKD). VDR responsive genes are involved in several cellular functions, such as cell proliferation, differentiation, membrane transport, and oxidative stress. Increased oxidative stress promotes the generation of advanced glycosylation end products (AGE) and related receptors (RAGE), with circulating RAGE contributing to protective responses against cardiovascular and renal diseases. Interestingly, the authors evaluated several biomarkers of oxidative stress in CKD patients, including myeloperoxidase activity, which is a pivotal enzyme involved in endogenous oxidant production. In contrast to previous studies, the evidence that paricalcitol stimulation does not alter the AGE/RAGE system and myeloperoxidase in CKD patients is here for the first time provided.

A research article from C. Prata and colleagues shows the insulin mimetic effect of sweet glycosides extracted from the leaves of the plant *Stevia rebaudiana.* Using several approaches, the authors demonstrate that steviol glycosides hold antioxidant ability stimulating, similarly to insulin, glucose uptake through Glut4 via activation of PI3K/Akt pathway, thus underscoring the emerging role of phytocompounds in facing several chronic and metabolic disturbs related to oxidative stress.

In conclusion, this special issue provides insights into the crosstalk between oxidative molecules and membrane transport in health and disease. Amongst other, phytocompounds are proposed as novel therapeutic approach to face oxidative stress-related pathological conditions.

